# Draft genome sequence of a triple hybrid *Escherichia coli* strain isolated from a healthy donor feces

**DOI:** 10.1128/mra.00113-24

**Published:** 2024-03-26

**Authors:** Judith Z. Ortega-Enríquez, Margarita M. P. Arenas-Hernández, Edwin Barrios-Villa

**Affiliations:** 1 Laboratorio de Biología Molecular y Genómica, Departamento de Ciencias Químico Biológicas y Agropecuarias, Universidad de Sonora, Campus Caborca. Av. Universidad e Irigoyen S/N, H. Caborca, Sonora, Mexico; 2 Posgrado en Microbiología, Centro de Investigaciones en Ciencias Microbiológicas, Benemérita Universidad Autónoma de Puebla, Ciudad Universitaria, Puebla, Mexico; Department of Biological Sciences, Wellesley College, Wellesley, Massachusetts, USA

**Keywords:** *Escherichia coli*, genomes, hybrid pathogen, diarrheagenic pathotypes

## Abstract

Herein is reported the draft genome sequence of a triple hybrid *Escherichia coli* strain isolated from a healthy donor feces. The assembly is 5.2 Mbp, composed of 247 contigs, with a N_50_ of 77, 241 bp, presenting a GC content of 50.8%.

## ANNOUNCEMENT

The relevance of *Escherichia coli* either as a commensal or as a pathogen has been widely documented (1). Pathogenic strains are classified as diarrheagenic (DEC) or extraintestinal (ExPEC) (2–5). Six DEC variants have been characterized and are well recognized, enteroaggregative (EAEC), enteroinvasive (EIEC), enterohaemorrhagic (EHEC), diffusely adherent (DAEC), entreotoxigenic (ETEC), and enteropathogenic *Escherichia coli* (EPEC) (6).

*E. coli* Ec-36.3 belongs to a strain collection previously characterized (7). It was isolated from a healthy donor feces in Caborca, Sonora, Mexico in October 2022. A stool sample from the healthy donor collected in a sterile container was diluted in 5 mL of saline solution, then 5 µL was plated onto a MacConkey (Becton Dickinson, BBL) agar plate and incubated aerobically at 37°C overnight. A lactose-positive colony was selected. This strain was characterized with biochemical tests using a Vitek2 system (Biomerièux, France) and molecularly using specific primers for *ybbW* as previously reported (Table 1) (8, 9); in addition, using a PCR approach were identified the genetic determinants of three diarrheagenic pathotypes: *bfpA* (but not *eae*, classifying it as atypical EPEC or aEPEC), *staI* (from ETEC), and *daaE* (from DAEC) (7) that codify for main subunit of bundle forming pilus, thermostable toxin, and the structural subunit of the F1845 fimbriae, respectively (Table 1). The strain was stored at −80°C in Luria Bertani (LB) broth supplemented with glycerol 20%. The sampling was carried out in accordance with the Declaration of Helsinki and a signed informed consent form was obtained from the participants.

**TABLE 1:** Specific primers and PCR conditions used for molecular characterization of Ec-36.3^*a*^

Target	Sequence (5′ – 3′)	Tm (amplicon size)	Reference
*ybbW*	F: gtgattggcaaaatctggccg	60°C (667 bp)	(8, 9)
	R: catactggcaatcagtacgcc		
*bfpA*	F: aatggtgcttgcgcttgctgc	67°C (326 bp)	(10)
	R: gccgctttatccaacctggta		
*eaeA*	F: caggtcgtcgtgtctgctaaa	67°C (570 bp)	(11)
	R: tcagcgtggttggatcaacct		
*staI*	F: ttaatagcacccggtacaagcagg	60°C (147 bp)	(12)
	R: cttgactcttcaaaagagaaaattac		
*daaE*	F: tgactgtgaccgaagagtgc	48°C (380 bp)	(13)
	R: ttagttcgtccagtaaccccc		

^*a*^ All the reactions were performed using 5µL of GoTaq MasterMix (containing reaction buffer, 400 µM of each dNTP, 3 mM MgCl_2_, and Taq DNA polymerase 1x) (Promega Corporation, USA), 0.3 µL of 25 mM of each primer and 1 µL of template DNA to a final volume of 10 µL. The reaction was of one cycle of 94°C 4 min for denaturing, followed by 35 cycles of 94°C, 1 min; specific Tm, 30 s; and 72°C, 60 s; and one final cycle of extension of 72°C for 5 min.

For DNA extraction, the Ec-36.3 was plated on agar MacConkey plates and one lactose-positive colony was inoculated on 5 mL of LB broth and incubated at 37°C overnight. Genomic DNA was obtained using the Wizard Genomic DNA purification kit (Promega, USA). Illumina sequencing libraries were prepared using the tagmentation-based and PCR-based Illumina DNA Prep kit (Illumina, USA) and custom IDT 10 bp unique dual indices with a target insert size of 320 bp. Illumina sequencing was performed on an Illumina NovaSeq 6000 sequencer producing 2 × 151 bp paired-end reads. In the following analysis, default settings were used unless otherwise noted. FastQC (v0.11.9) was used for quality evaluation and Cutadapt (v4.4) for trimming and cleaning of the raw reads (14). Assembly was performed with SPAdes (v3.15.4) (15). Twenty-four contigs were removed as they were below 200 bp in length. The assembled genome was 5, 233, 542 bp in length, composed of 247 contigs, which displayed an N_50_ of 77, 241 bp and a GC content of 50.8%. The estimated sequencing depth was 80× with a coverage of 100% of the *E. coli* K12 reference genome according to the NCBI database pipeline.

## Data Availability

The draft genome of this *E. coli* Ec-36.3 Whole-Genome Shotgun project has been deposited at DDBJ/ENA/GenBank under the accession JAYMYX000000000. The version described in this paper is JAYMYX010000000. The project accession number is PRJNA1060348. The raw sequencing reads were deposited to the GenBank under the accession number SRR27871076.
